# Enlarging back mass on a 33-year-old man

**DOI:** 10.11604/pamj.2013.16.54.2841

**Published:** 2013-10-15

**Authors:** Mouna Bouaddi, Badredin Hassam

**Affiliations:** 1 Department of Dermatology and Venereology, CHU IbnSina, Rabat, Morocco

**Keywords:** Warts, papillomatosis, HPV

## Image in medicine

Warts are tumor due to the infection with human papilloma virus HPV. We report the case of a giant wart evolving in the back of a thirty-three year-old man, This had been present since he had 7 years-old, but had been growing rapidly for the two last years. His medical history was devoid of recurrent infections, tumor pathologies. The exam revealed a brown tumor on the right paravertebral region that measured 25cm/7cm. It had irregular borders but well delineated. The surrounding skin was unaffected. Examination of the lymph nodes revealed multiple bilateral adenopathies exceeding 2cm in diameter. The rest of the physical examination revealed no additional abnormalities. A biopsy revealed papillomatosis, hyperkeratosis with parakeratosis, and the presence of koilocytes. The diagnosis was a giant wart confirmed by PCR. Neoplastic pathology was put forward as a second option due to the large size of the lesion, the speed of development, the peripheral adenopathies, as well as impaired overall health. These symptoms prompted the surgical excision of the tumor for a histological analysis on multiple slices of the specimen; no neoplastic proliferation was found. The uniqueness of our observation lies in the size, location, and length of evolution, as well as the unexplained increase in activity in the two years prior to examination. He reported having been employed as a mason during these years and frequently worked topless in the open air carrying heavy sachets on his back. These conditions could explain the warts increased activity related to autoinoculation.

**Figure 1:**
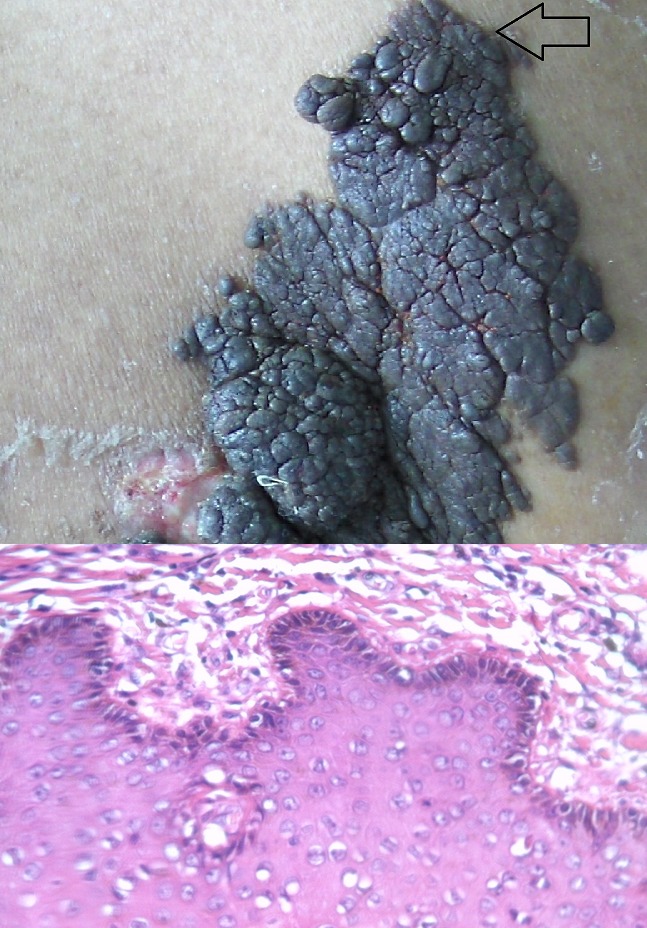
longitudinal verrucous tumor measuring 25cm x 7cm. Histology with HE coloration: epidermis with papillomatosis, hyperkeratosis and parakeratosis and presence of koilocytes

